# Tailored, psychological intervention for anxiety or depression in people with chronic obstructive pulmonary disease (COPD), TANDEM (Tailored intervention for ANxiety and DEpression Management in COPD): protocol for a randomised controlled trial

**DOI:** 10.1186/s13063-019-3800-y

**Published:** 2020-01-06

**Authors:** Ratna Sohanpal, Hilary Pinnock, Liz Steed, Karen Heslop Marshall, Claire Chan, Moira Kelly, Stefan Priebe, C. Michael Roberts, Sally Singh, Melanie Smuk, Sarah Saqi-Waseem, Andy Healey, Martin Underwood, Patrick White, Chris Warburton, Stephanie J. C. Taylor, On behalf of the TANDEM Investigators

**Affiliations:** 1 Institute for Population Health Sciences, Barts and the London School of Medicine and Dentistry, Queen Mary University of London, 58 Turner Street, London, E1 2AB UK; 2 Allergy and Respiratory Research Group, Usher Institute of Population Health Sciences and Informatics, Doorway 3, Medical School, Teviot Place, Edinburgh, EH8 9AG UK; 3 Newcastle upon Tyne NHS Hospitals Foundation Trust, Chest Clinic, New Victoria Wing RVI Hospital, Queen Victoria Road, Newcastle upon Tyne, NE1 4LP UK; 4 Department of Respiratory Sciences, College of Life Sciences, NIHR Leicester Biomedical Research Centre - Respiratory, Glenfield Hospital, University of Leicester, Groby Road, Leicester, LE3 9QP UK; 5 King’s Health Economics, Institute of Psychiatry, Psychology and Neuroscience, King’s College London, De Crespigny Park, Denmark Hill, London, SE5 8AF UK; 6 Warwick CTU, Warwick Medical School, Gibbet Hill Road, Coventry, CV4 7AL and University Hospitals of Coventry and Warwickshire, Clifford Bridge Road, Coventry, CV2 2DX UK; 7 School of Population Health and Environmental Sciences, King’s College London, Great Maze Pond, London, SE1 1UL UK; 8 London, UK

**Keywords:** COPD, Co-morbidity, Depression, Anxiety, Cognitive behavioural approach, Pulmonary rehabilitation, Complex intervention, Pragmatic randomised controlled trial, Clinical effectiveness, Health economic evaluation

## Abstract

**Background:**

People with chronic obstructive pulmonary disease (COPD) are at increased risk of depression and anxiety, which greatly reduces their quality of life and is associated with worse outcomes; but these psychological co-morbidities are under-recognised and undertreated in COPD patients. Pulmonary rehabilitation (PR) improves mood for up to 6 months but health practitioners under-refer, and patients commonly fail to attend/complete PR. Research suggests that complex non-pharmacological interventions, including both psychological and exercise components, may reduce anxiety and depression in COPD.

We have developed a tailored, cognitive behavioural approach (CBA) intervention for patients with COPD and co-morbid anxiety and/or depression (‘TANDEM’), which precedes and optimises the benefits of currently offered PR. We hypothesise that such a psychological intervention, delivered by supervised, trained respiratory healthcare professionals, will improve mood in patients with mild to moderate anxiety and/or depression and encourage uptake and completion of PR.

**Methods:**

We will conduct a multi-centre, pragmatic, randomised controlled trial of the TANDEM intervention compared to usual care across the Midlands, London, the South East and Bristol, UK.

We will train healthcare professionals familiar with COPD to deliver the manualised, tailored, face-to-face, one-to-one intervention weekly for 6–8 weeks.

We will recruit 430 participants from primary, community and secondary care with confirmed COPD and moderate to very severe airflow limitation, who are eligible for assessment for PR, and who screen positive for symptoms of mild/moderate depression and/or anxiety using the Hospital Anxiety and Depression scale (HADS). Participants will be randomised 1.25:1 (intervention: usual care).

The co-primary outcomes are the HADS anxiety and depression subscale scores at 6 months; participants will be followed up to 12 months. Secondary outcomes include uptake and completion of PR and healthcare resource use. There will be a parallel process evaluation and a health economic evaluation.

**Discussion:**

The TANDEM intervention has the potential to optimise the unrealised synergy between a psychological intervention and PR. The CBA sessions will precede PR and target individuals’ cognitions, behaviours and symptoms associated with anxiety and depression to decrease psychological morbidity and increase effective self-management amongst patients with COPD.

**Trial registration:**

ISRCTN, ID: ISRCTN59537391. Registered on 20 March 2017. Protocol version 6.0, 22 April 2018.

## Background

Chronic obstructive pulmonary disease (COPD) is an important public health problem associated with substantial morbidity and mortality [1–3] and a global prevalence of 11.7% in adults aged over 30 years [1]. People with COPD typically have multimorbidity, including psychological problems [4–6], such as anxiety and depression, which have a major influence on their quality of life and are associated with worse survival [7, 8]. Anxiety is reported across all ranges of COPD severity, with cited prevalence ranging from 10 to 50% [9, 10]. The prevalence of depression (typically around 30% of all COPD patients [9, 11]) increases with the severity of COPD [12]. Psychological problems are associated with lower levels of self-efficacy, persistent smoking, impaired health status and worse physical functioning [13, 14]. Additionally, people with anxiety/depression experience more exacerbations, more frequent and longer hospital admissions, and reduced survival [10, 13, 15–17]. The individuals’ disease may also affect the lives of their carers [18] creating a huge healthcare and economic burden [19, 20].

Despite effective interventions, co-morbid anxiety and depression are under-recognised and undertreated [21, 22]. This may be due to a number of reasons. A dominant medical culture results in very little attention to the patient’s psychological well-being, in spite of its impact in predicting disability and other outcomes [23]. Specific issues include healthcare professionals feeling ill-equipped to deal with emotional difficulties resulting from physical illness, stigma relating to the use of psychiatric or psychological services, and interpretation by patients that referral for psychological therapy undermines the validity of their symptoms. Pulmonary rehabilitation (PR), a well-recognised, evidence-based intervention for the management of COPD [24], reduces anxiety and depression [25] at up to 6 months’ follow-up but practitioners under-refer, and patients commonly fail to attend, or complete, their PR course [26, 27].

Cognitive behavioural therapy (CBT) improves anxiety/depression and is now recommended in guidelines for depression associated with long-term conditions [23], although a large, observational study of the effectiveness of routine, stepped-care psychological therapies for patients with long-term conditions in the UK found that they did less well than people without long-term conditions (controlling for co-variates) and that patients with COPD were amongst those with poorest outcomes [28]. A systematic review of psychological interventions for COPD identified four studies (total 193 participants) of CBT in people with COPD compared to usual care or education [29]. Although meta-analysis tended to favour the intervention both for anxiety and depression, the results were not statistically significant and the authors called for: ‘further research through well-designed and appropriately powered studies before inclusion in COPD guidelines or clinical care.... Furthermore, research is required to determine the most appropriate setting for the delivery of effective psychological interventions to ensure maximum benefit to patients with COPD.’

A 2013 systematic review of single or multi-component intervention studies that included either psychological and/or lifestyle interventions in patients with COPD reported significant, moderate treatment effects on anxiety and depression only in those interventions which *also included* exercise components [30]. However, there was great heterogeneity within the included studies, different follow-up periods were combined, and only five of 29 included studies were directed at people identified as anxious or depressed at baseline.

Building on this research we developed a tailored, psychological cognitive behavioural approach (CBA) intervention for patients with COPD and co-morbid anxiety and/or depression (referred to as the TANDEM intervention), which precedes, links into and optimises the benefits of currently offered PR. We hypothesised that such a psychological intervention, delivered by supervised, trained respiratory healthcare professionals who are very familiar with COPD, would improve mood in people with mild to moderate anxiety and depression.

This protocol describes a pragmatic, randomised controlled trial (RCT) with a parallel health economic evaluation which will assess whether delivery of the TANDEM intervention *prior* to routine PR improves anxiety and/or depression and is cost-effective in people with COPD with moderate to very severe airflow limitation and mild to moderate anxiety and/or depression. The RCT will also assess whether the intervention encourages attendance and completion of PR.

The protocol has been written following the Standard Protocol Items: Recommendations for Interventional Trials (SPIRIT) guidance [31] (see Additional file 1 populated SPIRIT Checklist).

### Aims and objectives

The primary aim is to evaluate a tailored, psychological cognitive behavioural approach (CBA) intervention (referred to as the TANDEM intervention), which precedes, links into and optimises the benefits of currently offered PR, with the aim of reducing mild/moderate anxiety and/or depression symptoms in people with COPD and moderate to very severe airflow limitation, as classified in the COPD GOLD guidelines [32].

The specific objectives are:

1. To undertake a RCT of the TANDEM intervention to examine the effectiveness of this intervention on clinical outcomes compared to usual care (i.e. the offer of PR alone)
2. To examine the effect of the TANDEM intervention (which is directed at patients) on their carers (where appropriate)
3. To determine the cost-effectiveness of the TANDEM intervention from a National Health Service (NHS) and personal social services perspective
4. To conduct a process evaluation to inform the implementation of the TANDEM intervention if the trial is positive, or assist interpretation of findings if it is negative

### Trial design

This is a multi-centre, parallel-group, pragmatic, randomised controlled, superiority trial including an internal pilot. The unit of randomisation is the individual study participant. We will also undertake a health economic evaluation and a process evaluation. Throughout the study and intervention design, delivery and assessment we have considered the National Institute of Health Behavioural Change Consortium’s treatment fidelity framework [33, 34].

Public involvement, in the form of patients with COPD and carers, are involved in the design and implementation of the trial to improve the relevance and overall quality of the research [35].

## Methods

### Setting and participants

Recruitment sites include PR services across a wide range of urban, suburban and rural areas: London, outer London (Berkshire, Wessex), the Midlands (Birmingham, Coventry, Leicester, Warwick) and Bristol, plus primary care, community care and secondary care settings associated with (i.e. able to refer patients to) these services, (a full list is available from the authors).

Eligible participants are adults with a confirmed diagnosis of COPD with moderate to very severe airflow limitation (Global Obstructive Lung Disease (GOLD) criteria) who are eligible [32] to be *assessed* for PR at their local service and, on screening, whose Hospital Anxiety and Depression Scale (HADS) scores are suggestive of mild to moderate: anxiety, depression or both (i.e. depression or anxiety subscale scores in the range ≥ 8 to ≤ 15 [36]). Patients who are receiving a psychological intervention, or who have received this within the preceding 6 months, are excluded but those taking prescribed medication for anxiety or depression remain eligible. Participants will also be invited to suggest a carer we could approach to include in the study to examine the effect of the patient-directed intervention on their carers. A full list of inclusion and exclusion criteria is provided in Table 1.

**Table 1:** Study inclusion and exclusion criteria

Inclusion criteria	Exclusion criteria
Patients	
• Adults with a confirmed diagnosis of COPD, post-bronchodilator FEV_1_/FVC ratio < 70% on spirometry • Moderate, severe or very severe COPD severity on spirometry, FEV_1_ < 80% predicted • Probable mild/moderate anxiety and/or depression as determined by the HADS-A and/or HADS-D scores ≥ 8 to ≤ 15 • Eligible to attend assessment appointment at their local pulmonary rehabilitation service at the time of randomisation i.e. 12 months have elapsed since last undertook PR or participant has another indication for PR referral (e.g. recent deterioration; recent hospitalisation with an acute exacerbation of COPD) [31] (Patients who have been offered PR previously but declined the offer or did not complete PR will be included)	• Unable to give valid consent • Patients with both HADS-A and HADS-D score < 8 (within normal range) • Severe anxiety/depression suggested by HADS-A or HAD-D score > 15 • If a patient has an appointment to commence PR < 4 weeks after the screening visit (because there is insufficient time to receive the TANDEM CBA intervention prior to PR starting) • Ineligible for PR at their local service at the time of randomisation (e.g. < 12 months since undertaking a course of PR and no new clinical indications [31] • A co-morbidity so severe that it would prevent the patient from engaging fully in the intervention and/or trial processes. (including: severe uncontrolled psychological or psychiatric disorder; moderate/severe cognitive impairment) • In receipt of a psychological intervention primarily directed at helping to manage anxiety or depression in the last 6 months (NB those taking antidepressants/anxiolytics not excluded) • Patients currently involved in another clinical trial related to COPD (to avoid over-burdening participants) • Insufficiently fluent in English to be able to complete the intervention and/or questionnaires
Carers	
• Identified by a participant as a ‘particular family caregiver or friend who helps them’ whom they would be happy for us to invite to join the study	• Unable to give valid consent • Not sufficiently fluent in English to be able to complete the questionnaires

Abbreviations: *COPD* chronic obstructive pulmonary disease, *FEV*_*1*_ forced expiratory volume in 1 second, *FVC* forced vital capacity, *HADS* Hospital Anxiety and Depression Scale (*D* Depression subscale, *A* Anxiety subscale) [34]; *PR* pulmonary rehabilitation

### Intervention and comparator

#### Intervention group

The intervention is delivered on a one-to-one basis by ‘TANDEM Facilitators’ who are specially trained healthcare professionals experienced in working with people with COPD (physiotherapists, nurses, occupational therapists or psychologists). To avoid any risk of contamination by inadvertently exposing control group patients to the TANDEM intervention content or ethos, members of staff delivering PR in participating centres are not eligible to deliver the TANDEM intervention.

We will describe the development of the intervention and the facilitator training in detail elsewhere (in preparation). Briefly, the TANDEM intervention is a tailored, manualised intervention which draws on: the principles of cognitive behavioural therapy (CBT) recommended for the treatment of anxiety and depression [23] in physical conditions including COPD [37–40]; our previous work on The Lung Health Manual for anxiety in COPD [41]; and self-regulation theory [42, 43]. Sessions are reinforced with written and DVD-based materials (TANDEM-specific hand-outs, leaflets and a DVD provided by the British Lung Foundation (see below) and self-management leaflets adapted from the SPACE manual [44], as indicated. Throughout the delivery of the intervention there is an emphasis on the links between symptoms (in particular breathlessness), thoughts, feelings and behaviours. The intervention aims to promote: techniques to manage breathlessness and to increase physical activity; to reduce social isolation; and to prepare for PR. Following the individual sessions, and if they have met the local PR assessment criteria, the participant has the opportunity to commence routine PR at their local service.

The intervention is delivered face to face in the participant’s home, or at a nearby NHS venue of their choice, weekly for 6 to 8 weeks, depending upon the severity of their initial anxious or depressive symptoms, or the presence of symptoms of both conditions and the patient’s individual progress. Sessions last for 40 to 60 min. On completion, with the participant’s permission, a written case summary is provided to their healthcare providers to document progress and highlight any need for further support. Between completing the TANDEM face-to-face intervention and up to 2 weeks after completing PR, facilitators also offer very brief (15 min or less), weekly or less frequent (depending on participant preference), CBA telephone support.

TANDEM Facilitators themselves receive regular telephone supervision by an experienced clinical psychologist, or an experienced cognitive behavioural therapist, trained in the supervision of TANDEM Facilitators. Facilitators receive telephone supervision at set times during the delivery of the intervention for each patient (typically, 15 min supervision after the second, third and fifth weekly session for each participant receiving a 6-week intervention.) Ad hoc telephone or email support from supervising psychologists and the TANDEM chief investigators is available throughout the study.

With participant permission, each face-to-face TANDEM session will be digitally recorded on an encrypted voice recorder. Trained health psychology coders will rate up to two, randomly selected, complete sets of intervention audio recordings per facilitator. The entire face-to-face TANDEM intervention as delivered to the participant will be rated for therapeutic competence and adherence [45, 46]. To assess the fidelity of intervention delivery psychologists experienced in health research will design a checklist which incorporates the Cognitive First Aid Rating Scale [45] and adherence to TANDEM content; specifically including both activities which should happen *repeatedly* (such as feedback on home practice), and *session-specific* activities (such as explaining intervention aims in Session 1).

We will be alert to the possibility that, due to the progressive nature of COPD, there is potential for patient participants to become increasingly distressed by their situation and their physical condition. We do not envisage this happening but there is a small risk that some participants may become much more anxious or depressed, or (very unlikely but more seriously) may express suicidal intent such that they are at risk of harm to themselves or to others. Throughout the study, the CBA facilitators will all receive ongoing supervision from a senior clinical psychologist/nurse consultant (trained in CBT) to help them identify and respond appropriately to this possibility. The development of much worse depressive or anxious symptoms, or suicidal ideation, would be criteria for discontinuing the allocated (CBA) intervention. These will be reported as adverse events which will be recorded and reported in line with the Ethics Committee’s and study sponsor’s requirements.

#### Control group

Participants in the control arm receive usual care and will be referred to their local PR service in the usual way. They will be offered the usual multidisciplinary PR programme provided in their local area (including any psychological treatment provided routinely in that service). Because there is usually a delay between referral and commencement of PR courses (in 2017 the median waiting time was around 11 weeks [47]), and we are not enrolling participants where the average waiting list for PR is less than 4 weeks, control group participants will attend PR at around the same time as the intervention group.

In addition, all participants also receive a publicly available British Lung Foundation (BLF) DVD: ‘Living with COPD’/‘Stay Well Stay Active’ and a publicly available BLF COPD information and exercise and PR booklet to ensure best usual care.

### Outcomes

There is no agreed core outcome data set for trials in COPD (http://www.comet-initiative.org). We selected the HADS anxiety and depression subscales (HADS-A and HADS-D, respectively) [36] as our co-primary outcome measures since eligible participants may have either (or both) conditions. A recent review of instruments to screen for depression in COPD examined the four most commonly used measures (including HADS) and concluded that there was no evidence that any instrument was superior [11] but noted that HADS is the only measure specifically validated in COPD patients [48]. All outcome measures are collected at baseline, 6 and 12 months following randomisation. We elected to measure primary outcomes at 6 months because we hypothesised that our intervention would be effective well within that timescale [30].

Secondary outcomes include: the Beck Anxiety Inventory [49] and the Beck Depression Inventory II [50, 51]; respiratory-related quality of life using the St George’s Respiratory Questionnaire [52]; the Brief Illness Perception Questionnaire [53]; social engagement (University of Melbourne Health Education Impact Questionnaire social engagement scale [54]); the EuroQol instrument EQ-5D-5 L [55]; attendance at, and completion of, PR; and smoking status. Social functioning will be measured using an adapted version of the United Kingdom Time Use Survey [56], which lists potential activities and asks participants how many times they had engaged in the activity in the week prior to assessment, how long was spent doing the activity and whether this was done alone or accompanied by with another person [57]. We will also collect all healthcare resource use during the 12 months of follow-up. From participating carers we will collect the Zarit Caregiver Burden Inventory [58] and the Warwick Edinburgh Mental Well-Being Scale [59].

Table 2 shows all the study variables and data collected from the study participants and carers. In addition, data will be collected on adverse events and/or serious adverse events related to study procedures. 
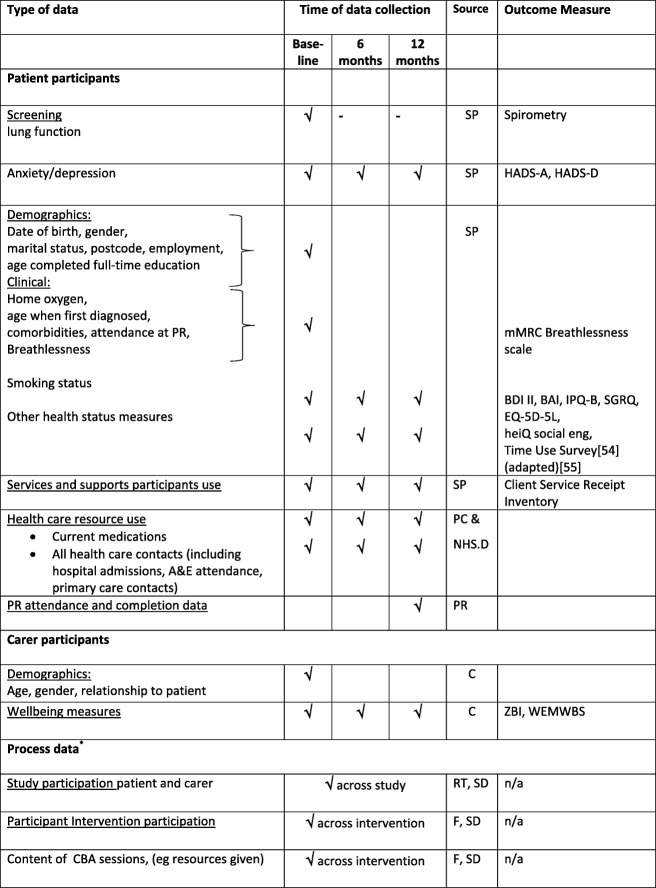

**Table 2:**
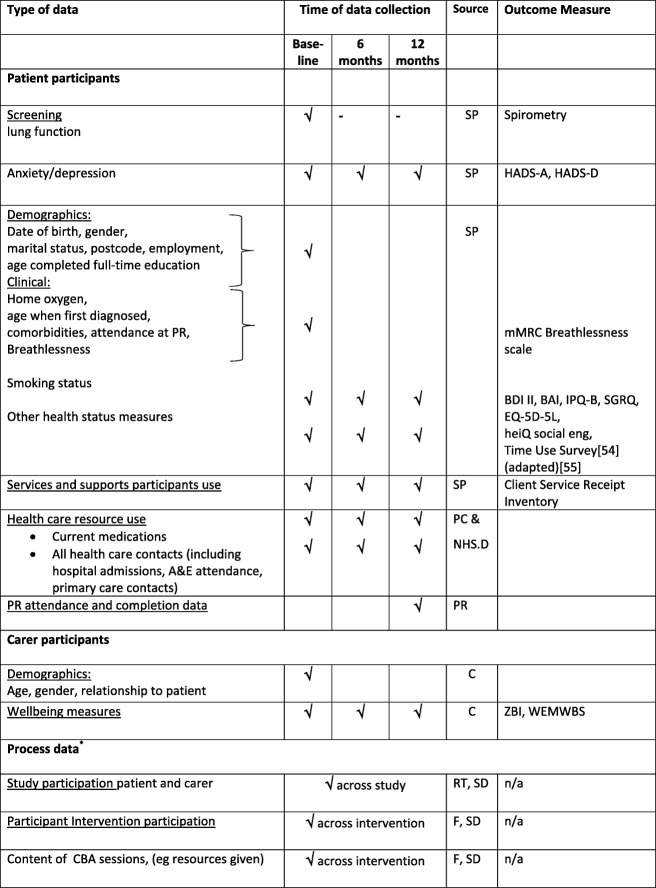
Study data collection

Abbreviation and footnotes: Sources: SP = study participant, PC = primary care medical records, NHD.D = NHS Digital, PR = pulmonary rehabilitation services, C = carers, RT = research team, F = TANDEM facilitators, SD = study documentation. Outcome measures: HADS = Hospital Anxiety and Depression Scale (D = depression sub-scale; A = anxiety subscale) [34]; mMRC = modified MRC breathlessness scale [61]; BAI = Beck Anxiety Inventory [47]; BDI II = Beck Depression Inventory II[48]; SGRQ = St George’s Respiratory Questionnaire [50]; heiQ social eng = University of Melbourne Health Education Impact Questionnaire social engagement scale [52]; IPQ-B = The brief illness perception questionnaire [51]; EQ-5D-5L = The EuroQol instrument [53]; ZBI = Zarit Caregiver Burden Inventory [56]; WEMWBS= Warwick Edinburgh Mental Well-Being Scale [57]; n/a = not applicable.

*process evaluation interview data not included here.

### Recruitment and study procedures

All study participants will provide written informed consent and are aware that they can withdraw from the study at any time. Potential participants will be recruited through advertisements in secondary care and community clinics or approached by clinical or clinical research staff and asked if the study researcher may contact them to discuss participation. We will also invite general practices proximate to selected participating PR centres to screen their records for people who may be eligible and to contact them by post. Potential study participants who are interested will be assessed in the same manner as those recruited from secondary or community care.

The study researcher will arrange spirometry and administer the HADS to confirm eligibility. Eligible participants will then be recruited to the study and baseline data will be collected before randomisation to intervention or control. Allocation will be concealed by use of a remote, computer-generated randomisation service based at the Pragmatic Clinical Trials Unit (PCTU) (https://www.qmul.ac.uk/pctu/).

All patient and carer study participants will be followed up in a location chosen by the participant (home, GP practice, local hospital or community clinic) at 6 and 12 months by a study researcher. Baseline and outcome data are collected as supervised, self-completed questionnaires with provision for postal data collection if requested by participants, and telephone follow-up for primary outcomes only if participants fail to complete questionnaires. With the exception of the visual analogue scale of the EQ-5D-5 L (which has to be recorded on paper), participants will input questionnaire data directly using OpenClinica software via a study tablet computer (with 3G/4G connection) unless they prefer to use a paper version of the questionnaire. Researchers collecting data are blind to the allocation arm of participants, who are asked not to disclose their treatment allocation to researchers.

All study data will be uploaded onto a dedicated folder on the secure virtualised environment at the Barts Cancer Centre (BCC), Queen Mary University of London (trial sponsor). This is where all data analysis of the PCTU trial data is carried out. The BCC environment requires dual-factor authentication to access the portal and the folders where the data are stored are only accessible to the appropriate members of the PCTU and the TANDEM study team.

**Fig. 1:**
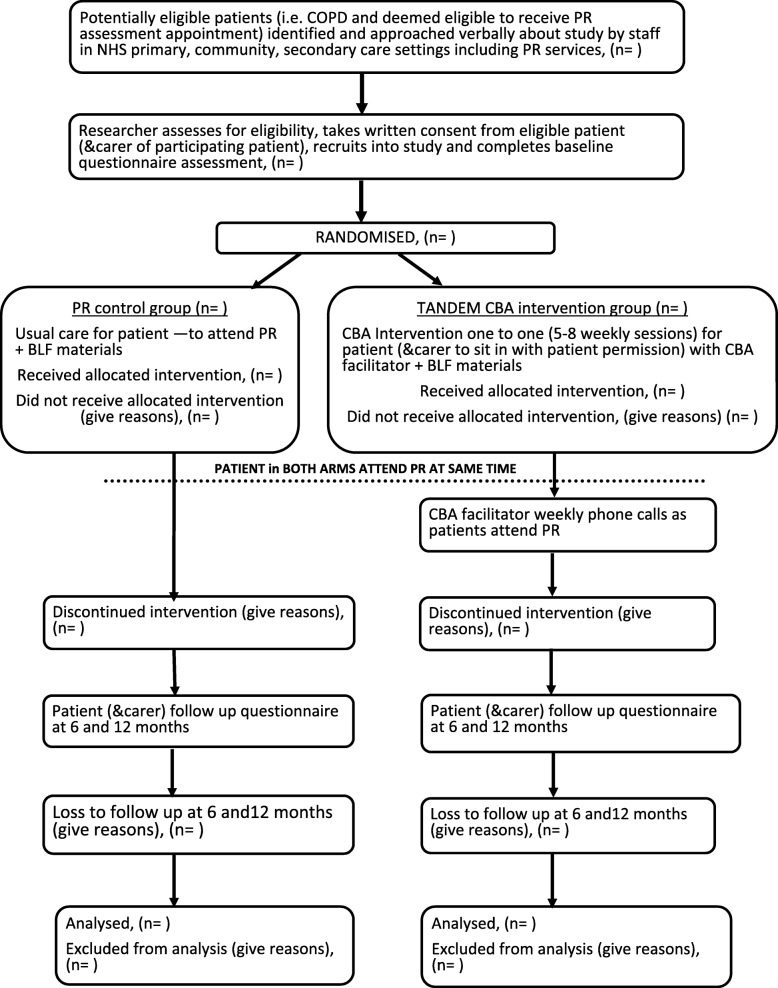
Study flow diagram

**Fig. 2:**
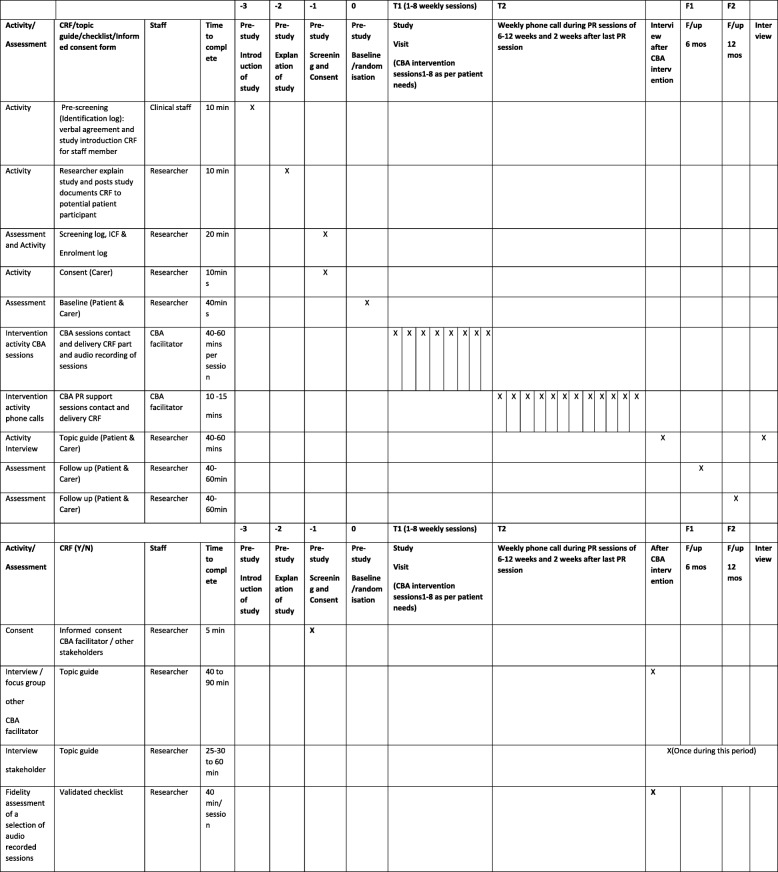
Standard Protocol Items: Recommendations for Interventional Trials (SPIRIT) Figure: study visits, activity and assessments of patient and carer participants

Figure 1 shows the study flow diagram. Figure 2 (SPIRIT Figure) provides information on the study visits and the activities/assessments at each visit. A process evaluation and cost-effectiveness analysis will be performed.

A pilot RCT (*n* = 45) was completed in 2018. Data from this will be incorporated as an internal pilot as no significant changes were made to the intervention or to study procedures.

### Sample size

Sample size calculations are based on the co-primary outcomes: HADS-A (anxiety) and HADS-D (depression) at 6 months. Based on a significance level of 2.5% and 90% power, recruiting 153 participants per arm (306 in total) would allow us to detect a difference of 1.7 points on the HADS-A subscale, and 1.5 points on the HADS-D subscale (based on an SD of 4.2 for anxiety and 3.6 for depression [61]); these are equivalent to a standardised mean difference of about 0.4.

In trials where there is clustering in one arm only, power is maximised by using an unequal allocation ratio favouring the group with clustering [60]. The exact allocation ratio should be similar to the design effect used to inflate the sample size. Due to the clustering effect by Facilitator in the intervention arm, we increased the sample size. Assuming an intra-class correlation coefficient between facilitators of 0.01 contributing to the primary outcome, and 24 participants per therapist at follow-up, leads to a design effect of 1.23, which requires increasing the number of participants in the intervention arm to 189 (342 overall). Assuming a study dropout rate of 20%, we would require 428 participants overall. This has been rounded up to 430. Using an allocation ratio of 1.25 vs. 1, this will lead to approximately 240 participants in the intervention arm and 190 in the control arm.

### Randomisation

Randomisation will be stratified by NHS Trust and minimisation used within each stratum with a random element in order to minimise potential imbalances at baseline for anxiety (HADS-A), depression (HADS-D), breathlessness (mMRC – modified Medical Research Council scale [62]) and smoking status.

### Statistical analysis

Analyses will be by intention-to-treat (ITT): i.e. we will analyse all participants, for whom an outcome is available, according to the treatment group to which they were randomised. All analyses will account for clustering by facilitator in the intervention arm, and each analysis will present a treatment effect (difference in means for continuous outcomes, odds ratios for binary outcomes) with a 95% confidence interval and a two-sided *p* value. Outcomes at 6 and 12 months will be analysed using a mixed-effects regression model that will account for correlation within TANDEM Facilitators, and correlation between outcomes at 6 and 12 months. Analyses will adjust for the outcome measured at baseline when possible. We will use a Hochberg procedure [63] to analyse the two primary outcomes. Briefly, the Hochberg procedure states that if either outcome has a *p* value < 0.025 then that outcome is statistically significant; additionally, both outcomes are significant if the *p* values are both < 0.05.

We will perform various sensitivity analyses. We will assess the impact of any difference in time from baseline to attending PR in intervention and control groups. Because our inclusion criteria states that participants must have a score of ≥ 8 on either the HADS-A or HADS-D subscale (but not necessarily both), some participants will score < 8 on one of these subscales and have less room for improvement during follow-up. We will assess the impact of including such participants.

We will also perform sensitivity analyses to assess the robustness of our results to various assumptions regarding missing data, or participants lost to follow-up: for this we will assess the feasibility of using a multiple imputation approach (depending on the entity and the structure of missingness). A detailed Statistical Analysis Plan will be prepared by the PCTU statisticians and signed off prior to unblinding.

### Economic evaluation

The economic analysis will assess whether the addition of a tailored psychological intervention, combined with the availability of standard PR, is likely to be a cost-effective use of resources. The economic evaluation will take a NHS and Personal Social Services Perspective as currently preferred by the National Institute for Health and Care Excellence (NICE) [64]. TANDEM sessions (both face to face and telephone contact) and TANDEM Facilitator support will be recorded centrally. The economic evaluation will be carried on an ITT basis (as per the ‘Statistical analysis’).

Patient contact with PR will be extracted from clinical records. Other community and hospital-based NHS and social care service contacts will be recorded via patient self-report using a version of the Adult Service Use Schedule (AD-SUS) [65], supplemented where possible with administrative data through primary care or NHS Digital sources. Unit costs for costing patient contact with the intervention will be developed using standard health economic methodology [66]. Unit costs for PR and other health and social care services will be extracted from published sources including the Unit Costs of Health and Social Care [66] and national NHS reference costs [67].

Total health and social care costs will be compared between the groups using a regression model which controls for baseline co-variates. Cost data are usually skewed and so bootstrapped confidence intervals will be generated around the cost difference. Incremental cost-effectiveness will be evaluated based on the primary outcome measures and on quality-adjusted life years gained (QALYs). The latter will be derived from the EQ-5D-5 L using area-under-the-curve methods [68]. Point estimates of population mean cost and outcome differences between the groups will be used to produce incremental cost-effectiveness ratios (ICERs). Uncertainty around cost-effectiveness estimates on account of trial sampling error will also be assessed using cost-effectiveness acceptability curves and presentation of the distribution of cost and outcome pairings within the cost-effectiveness plane generated from bootstrap trial-data simulations. Deterministic sensitivity analysis will also be used to assess the sensitivity of cost-effectiveness conclusions to changes in assumptions made regarding relevant economic parameters. Any missing economic data will be replaced using a multiple imputations approach (subject to entity and structure of missingness).

The primary economic analysis will evaluate the within-trial cost-effectiveness of the TANDEM intervention over follow-up. However, given the possibility that the intervention could improve engagement with PR over the longer term, we will also explore the feasibility of adapting existing economic models of the impacts of COPD to provide extrapolations of longer-term benefits to the NHS and patients of increased PR engagement beyond the trial period.

A detailed Health Economic Analysis Plan will be prepared by the trial economists and signed off prior to unblinding.

### Process evaluation

A parallel-process evaluation [69] will be conducted. This will assist the implementation of the intervention if the trial is positive, or assist in the interpretation of findings if the study is negative. The process evaluation will be informed by Normalisation Process Theory [70, 71] and will include: collection of process data (e.g. number and nature of modules delivered to each intervention participant); qualitative interviews with study participants and carers, health care professional (HCP) facilitators delivering the intervention and their clinical psychologist supervisors, and a wide range of potential stakeholders including PR teams, service managers, commissioners, general practitioners and community psychology services; and assessment of the fidelity of the delivery of the intervention (described above). The full process evaluation will be described in detail elsewhere (in preparation).

## Discussion

The TANDEM intervention has the potential to optimise the unrealised synergy between a psychological intervention and PR. The CBA sessions will precede PR and target individuals’ cognitions, behaviours and symptoms associated with anxiety and depression to decrease psychological morbidity and increase effective self-management amongst patients with moderate to severe COPD. The hope is that this will also increase the likelihood of attending and completing PR which in itself has a positive effect on anxiety and depression, in addition to benefits on quality of life and exercise tolerance. The psychological and physical benefits of the TANDEM individual sessions and PR are thus synergistic, though even participants who do not engage with PR following the CBA sessions should benefit.

The intervention has been designed so that realistically it could be implemented across health services: screening people referred for PR for anxiety and depression and intervening before they start PR; being delivered by an existing group of health professionals who are familiar with the management of COPD and the day-to-day experiences of those living with advanced COPD; and linking into, and optimising, routine PR.

### Trial status

The trial is ongoing. Protocol version 6 dated 22 April 2018. The recruitment began on 14 June 2017 and is likely to be completed by 31 March 2020.

## Supplementary information

**Additional file 1.** Standard Protocol Items: Recommendations for Interventional Trials (SPIRIT) 2013 Checklist: recommended items to address in a clinical trial protocol and related documents*.

## Data Availability

Not applicable
